# Rational regional distribution of sugarcane cultivars in China

**DOI:** 10.1038/srep15721

**Published:** 2015-10-26

**Authors:** Jun Luo, Yong-Bao Pan, Liping Xu, Michael Paul Grisham, Hua Zhang, Youxiong Que

**Affiliations:** 1 Key Laboratory of Sugarcane Biology and Genetic Breeding, Ministry of Agriculture, Fujian Agriculture and Forestry University, Fuzhou 350002, Fujian, China; 2 USDA-ARS, Sugarcane Research Unit, Houma, LA 70360, USA

## Abstract

Knowing yield potential and yield stability of sugarcane cultivars is of significance in guiding sugarcane breeding and rationalising regional distribution of sugarcane cultivars. In the present study, a heritability-adjusted genotype main effect plus genotype × environment (HA-GGE) biplot program was used to analyze the cane and sucrose yields of 44 newly released sugarcane cultivars at eight pilot test sites. The cane and sucrose yields of nine cultivars were higher than those of the control cultivar ROC22. From the perspective of cane yield, cultivars FN 40 and YZ 06–407 were well adapted to a wider range of conditions and produced relatively high cane yields in several pilot sites. From the perspective of sucrose yield, cultivars LC 03–1137, FN 38, FN 41, MT 01–77 and LC 05–136 were well adapted to a wide range of conditions and produced relatively high sucrose yields. Based on these results, three high yielding and widely adapted cultivars, namely, FN 39, LC 05–136, and YZ 05–51 were recommended for production in three major Chinese sugarcane planting areas. The results will provide a theoretical basis for recommending the effective use and rational regional distribution of sugarcane cultivars in China.

In China, sugarcane accounts for more than 80% of total sugar production. During the period 1949 to 2013, the acreage of sugarcane planting increased 13.8 times from 0.124 million ha to 1.71 million ha, and sugar production increased 44.54 times from 0.2822 million tons to 12.5717 million tons. Sugarcane planting areas have also gradually shifted from 11 to three provinces in southern China, i.e., south central Guangxi, southwestern Yunnan, and western Guangdong-northern Hainan^1^. ROC22, ROC16, ROC25, and other ROC series cultivars from Taiwan account for about 75% of the total sugarcane acreage, which may lead to a genetic uniformity problem. With the development and release of a large number of sugarcane cultivars in recent years in China, including YT 93–159, GT 21, FN 15, FN 91–4621, FN 39, and YT 00–236, the genetic uniformity situation dominated by the ROC-series cultivars has been gradually improved^2^. Both plant cane and ratoon crops of 38 varieties have been evaluated through four rounds of integrated demonstrative experiments at 15 integrative experiment stations. At 14 province-administered regional pilot field sites, 10 rounds of preliminary variety tests have been conducted involving a total of 174 varieties. Of these varieties, 141 varieties were subject to nationwide regional variety trials and 89 varieties were undergone production tests. Seventy-three varieties have acquired national cultivar certificates. However, how to guide rational distribution of these cultivars based on their yield potential and yield stability to different sugarcane planting areas remains a difficult research topic.

Genotype and environment (G × E) interaction is the main factor that affects both yield potential and yield stability of crop varieties. The more interaction between genotype and environment, the less stable the variety would be. In terms of yield and other quantitative traits, yield differences due to G × E are far greater than those caused by variability among genotypes^3^,^4^,^5^. A large number of studies on G × E effect on crop growth, yield, and other agronomic traits have been conducted all over the world^6^, including wheat^7^, corn^8^,^9^, rice^10^, rape^11^,^12^, sugarcane^13^, peanut^14^, sunflower^15^, tea^16^, soybean^17^, sweet potato^18^, lentils^19^. Kang and Miller^20^ revealed that the instability of cane and sugar yields was introduced by the linear effect of the covariate. Besides, the repeatability of stability-variance parameters between crops (individual repeatability) was relatively low. Jackson *et al.*^21^ evaluated the response and association of sugarcane with environmental factors. They found that soil nutrient levels, or other related factors, might be responsible for family × environment interactions in sugarcane. Zhou *et al.*^22^ determined the variance components of G × E and evaluated their relative importance and implications in the breeding programmes. The results showed that the genotype by crop-year variance component was more significant for the rainfed than irrigated programmes, while genotype by location by crop-year interaction was more significant for yield than sucrose content. Besides, breeding and selecting for sugarcane ratooning ability were critical in rainfed regions, highlighting the complexity associated with sugarcane regional breeding. Additive main effects and multiplicative interaction (AMMI) and GGE biplot models are the two most popular statistical methods for data analysis in crop cultivar trials^23^,^24^. Bissessur *et al.*^25^ used AMMI to depict the sugarcane varieties for both wide and specific adaptations in Mauritius. The GGE biplot method uses plots to analyze diallel data. One key feature of the GGE biplot is to only display the genotype’s main effect (G) and G×E interaction. In addition, Yan used an average tester coordinate to simplify the GGE biplot by comprehensive display of information in a two-way data table^26^,^27^,^28^,^29^,^30^. Yan^24^ proposed the use of the GGE biplot method in regional wheat trials. Luo *et al.*^31^ applied the GGE biplot to analyze the data from seven-cultivar trials at seven pilot sites and identified two cultivars FN 39 and YZ 05–51 with relatively high yield and stability. Zhou *et al.*^32^ applied the GGE biplot to analyze experimental data from nine soybean strains at five pilot sites of Heilongjiang Province during 2008–2009 and found that strain Tender 0022111 exhibited high yield with good stability. Zhang *et al.*^33^ analyzed data from national oat regional trials during 2006–2008 and identified three oat strains with high and stable yields. HA-GGE is a heritability adjusted GGE biplot model^30^,^34^. Xu *et al.*^34^ used the HA-GGE biplot to evaluate the test environments among different cotton planting regions in the Yangtze River basin. They were able to divide the cotton planting regions into different ecological zones based on lint yield^35^.

Pilot demonstrations of sugarcane cultivars provide the basis for regional distribution and are of great importance in accelerating inter-provincial promotion of superior sugarcane cultivars, increasing the income of sugarcane farmers, and promoting the sustainable development of sugarcane industry. Currently, no evaluation has been conducted in China uses the HA-GGE biplot method on yield and yield stability of newly released sugarcane cultivars. In this study, the HA-GGE biplot method was used to the analyze yield trial data involving 44 newly released sugarcane cultivars and eight national pilot experimental sites. The yield trial experiments were designed to screen sugarcane cultivars for both high yield and high yield stability and to distribute top performing cultivars to the three major sugarcane production areas in China. In addition, the discriminating power and representativeness of the test environments were also assessed comprehensively.

## Results

### Testing for significant differences among the yields of different cultivars at different pilot sites

Analysis of variance showed that the pilot site environmental effect (E), genotype effect (G), GE interaction and the effect of test block within the pilot site were all significant (Table 1). Pilot site (E) was the most important factor affecting yield, accounting for 43.58% (cane yield) and 44.56% (sucrose yield) of the total variations. The next is GE interaction, accounting for 33.42% (cane yield) and 30.74% (sucrose yield) of total variations. Genotype (G) only accounts for a relatively small portion of the total variations: 13.35% (cane yield) and 13.80% (sucrose yield). The G/GGE effect for cane and sucrose yields are 0.29 and 0.31, respectively (Table 1). Therefore, the effect of GE was far greater than the G effect. Based on the two year yield data collected from the eight pilot sites and 45 genotypes (Table 1), the current average cane and sucrose yields in China are 98.65 Mg·ha^–1^ and 14.26 Mg·ha^–1^, respectively. This reflects the general yielding capability of newly bred Chinese sugarcane cultivars under current ecological and production conditions. Within the same crop season, however, the coefficients of variation between different pilot sites varied in the range of 10.20–20.71% for cane yield and 10.65–21.20% for sucrose yield (Table 2).

### Optimal regions of sugarcane cultivars

**Figure 1:**
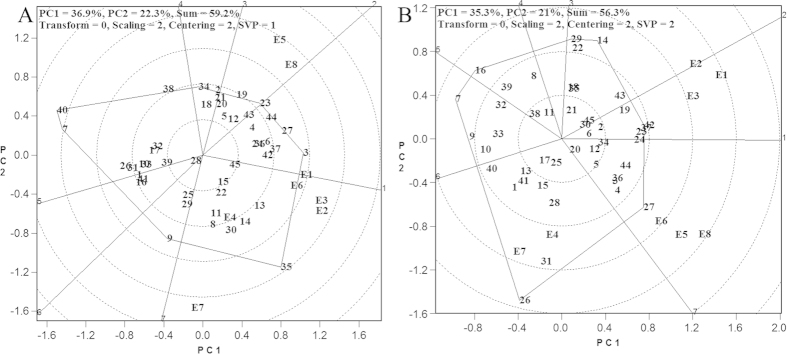
Adaptability of sugarcane cultivars based on HA-GGE biplot analysis. (**A**) cane yield; (**B**) sucrose yield; PC1: the first principal component; PC2: the second principal component; E1: GDZJ, Guangdong Zhanjiang; E2: GXBS, Guangxi Baise; E3: GXCZ, Guangxi Chongzuo; E4: GXLB, Guangxi Laibing; E5: GXLZ, Guangxi Liuzhou; E6: HNLG, Hainan Lingao; E7: YNDH, Yunnan Dehong; E8: YNLC, Yunnan Lincang; 1 = YZ 99–91; 2 = YZ 06–80; 3 = YZ 06–407; 4 = YZ 05–51; 5 = YZ 05–49; 6 = YZ 04–241; 7 = YZ 03–422; 8 = YZ 03–258; 9 = YZ 03–194; 10 = YZ 03–103; 11 = YZ 01–1413; 12 = YZ 06–189; 13 = YT 96–86; 14 = YT 60; 15 = YT 55; 16 = YT 00–318; 17 = YT 00–236; 18 = YG 42; 19 = YG 40; 20 = YG35; 21 = YG 34; 22 = YG 24; 23 = MT 01–77; 24 = LC 05–136; 25 = LC 05–129; 26 = LC 03–182; 27 = LC 03–1137; 28 = GT 97–69; 29 = GT 30; 30 = GT 29; 31 = GT 02–901; 32 = GT 02–467; 33 = GT 02–351; 34 = GN 02–70; 35 = FN 40; 36 = FN 39; 37 = FN 38; 38 = FN 36; 39 = FN 30; 40 = FN 28; 41 = FN 15; 42 = FN 41; 43 = FN 42; 44 = DZ 03–83; 45 = ROC22

High cane yielding cultivars were identified at different pilot sites based on the diagram of optimal regions for sugarcane cultivars. This, in turn, was based on cane yield in the HA-GGE biplot, in the sectors of FN 40 (35) and YZ 06–407 (3) (Fig. 1A). A relatively large number of pilot sites were fairly widely distributed, suggesting that these two cultivars adapted to a wide range, and their cane yields were high at four test sites, Guangdong Zhanjiang (E1), Guangxi Baise (E2), Guangxi Chongzuo (E3) and Hainan Lingao (E6). The cane yield of MT 01–77 (23) was good in Guangxi Liuzhou (E5) and also relatively high in Yunnan Lincang (E8), suggesting that MT 01–77 had a relatively high adaptability. The cane yields of FN 36 (38), YZ 03–422 (7) and FN 28 (40) were relatively low in all pilot sites. Four cultivars, namely, ROC22 (45), FN 30 (39), GT 97–69 (28) and YT 55 (15), were located inside the polygon close to the origin and were not sensitive to the environmental changes. A diagram of optimal regions for cultivars based on sucrose yield is shown in Fig. 1B. MT 01–77 (23), LC 05–136 (24), FN 38 (37) and FN 41 (42) were adapted to a wide range of environments, and also had relatively high cane yield in Guangdong Zhanjiang (E1), Guangxi Baise (E2) and Guangxi Chongzuo (E3). The sucrose yield of LC 03–1137 (27) was relatively high in Guangxi Liuzhou (E5), Hainan Lingao (E6) and Yunnan Lincang (E8); whereas LC 03–182 (26) had a relatively high sucrose yield in Guangxi Laibin (E4) and Yunnan Dehong (E7). On the other hand, the sucrose yields of YT 00–318 (16) and YZ 03–422 (7) were relatively low at all pilot sites. Ten cultivars, namely, YZ 04–241 (6), YZ 01–1413 (11), YZ 06–189 (12), YT 00–236 (17), YG 35 (20), YG 34 (21), LC 05–129 (25), GT 29 (30), FN 36 (38) and ROC22 (45), were placed inside the polygon close to the origin and were insensitive to the environmental changes, which was in accordance with the claim of breeders that sucrose yield is a relatively stable trait^2^.

### The stability of sugarcane cultivars

**Figure 2:**
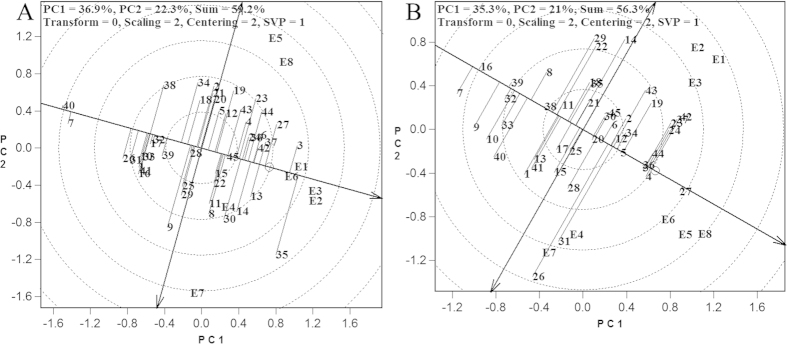
Yield performance and stability of sugarcane varieties based on HA-GGE biplot analysis. Refer to Fig. 1 for abbreviations.

To select sugarcane cultivars suitable for rational distribution, both high yield and yield stability are important. In Fig. 2A,B, the first principle component explained 36.9% (cane yield) and 35.3% (sucrose yield) of G + GE, whereas the second principle component explained 22.3% (cane yield) and 21% (sucrose yield) of G + GE. In total, the HA-GGE biplot explained 59.2% (cane yield) and 56.3% (sucrose yield) of G and GE effect. From the perspective of cane yield (Fig. 2A), 15 cultivars had greater than average cane yields in all the test environments that were also greater than the cane yield of the check cultivar ROC22. These 15 cultivars were FN 40, YZ 06–407, LC 03–1137, YT 96–86, FN 38, FN 41, YZ 04–241, YT 60, DZ 03–83, FN 39, LC 05–136, GT 29, MT 01–77, YZ 05–51 and FN 42. Among them, eight cultivars, namely, YZ 05–51, YZ 04–241, YZ 06–407, FN 39, LC 05–136, FN 38, FN 41 and YT 96–86, had cane yields that were not significantly affected by test sites. In contrast, the cane yields of the other seven cultivars (YT 60, GT 29, FN 40, MT 01–77, DZ 03–83, FN 42 and LC 03–1137) were less stable. Nineteen cultivars had sucrose yield that were higher than the average sucrose yields in all test environments and also higher than the sucrose yield of the control cultivar ROC22 (Fig. 2B). These 19 cultivars were LC 03–1137, FN 41, YZ 05–51, FN 38, LC 05–136, MT 01–77, DZ 03–83, YZ 06–407, FN 39, YG 40, YZ 05–49, GN 02–70, FN 42, GT 02–901, YZ 06–189, YZ 06–80, LC 03–182, YZ 04–241 and GT 97–69. Eleven of these 19 cultivars, namely, LC 05–136, YZ 06–80, YZ 04–241, GN 02–70, DZ 03–83, YZ 06–189, FN 39, YZ 05–49, YZ 06–407, LC 03–1137 and YZ 05–51 had relatively stable sucrose yields, whereas the other eight cultivars (GT 97–69, GT 02–901, LC 03–182, FN 42, FN 41, YG 40, FN 38 and MT 01–77) had relatively poor stability on sucrose yields.

### The representativeness of the test environments

**Figure 3:**
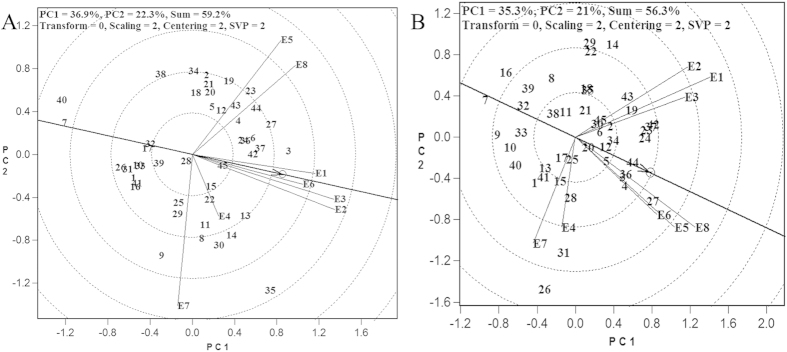
Discrimination power and representativeness of test environments based on HA-GGE biplot analysis. Refer to Fig. 1 for abbreviations.

In a biplot diagram, the length of the environment vector represents the discriminating power of the test environment, which is proportional to the vector length. The distance from the origin of the biplot to each icon of the test environment is the length of the corresponding environment vector. In terms of cane yield, the discriminating powers were high for Guangxi Baise (E2), Guangxi Chongzuo (E3) and Yunnan Dehong (E7); medium-high for Guangdong Zhanjiang (E1), Guangxi Liuzhou (E5), Hainan Lingao (E6) and Yunnan Lincang (E8); but low for Guangxi Laibin (E4) (Fig. 3A). In terms of sucrose yield, the discriminating powers were high for Guangdong Zhanjiang (E1), Guangxi Baise (E2), Guangxi Liuzhou (E5) and Yunnan Lincang (E8); medium-high for Guangxi Chongzuo (E3), Hainan Lingao (E6) and Yunnan Dehong (E7); and low for Guangxi Laibin (E4) (Fig. 3B).

The representativeness of test environment refers to its consistency with the average of all other test environments in the target region or the average of all test environments, and is represented by the angle between the environment vector and the average environment vector (Average environment coordination axis, AEC axis). The smaller the angle, the higher the representativeness. The AEC axis is the ray with origin as the initial point that connects the origin to the average environment icon, which is the average of the coordinates of all test environments^10^. From the perspective of cane yield, four pilot sites, namely, Guangdong Zhanjiang (E1), Guangxi Baise (E2), Guangxi Chongzuo (E3) and Hainan Lingao (E6), were highly representative environments. Three pilot sites [Guangxi Laibin (E4), Guangxi Liuzhou (E5) and Yunnan Lincang (E8)] were intermediately representative environments. Yunnan Dehong (E7) was the least representative of its target environment. From the perspective of sucrose yield, Guangxi Liuzhou (E5), Hainan Lingao (E6) and Yunnan Lincang (E8) were highly representative environments; Guangdong Zhanjiang (E1), Guangxi Baise (E2) and Guangxi Chongzuo (E3) were intermediately representative environments; and Guangxi Laibin (E4) and Yunnan Dehong (E7) were least representative environments. When combining both the cane and sucrose yield data, six pilot test sites, namely, Guangdong Zhanjiang (E1), Guangxi Baise (E2), Guangxi Chongzuo (E3), Guangxi Liuzhou (E5), Hainan Lingao (E6) and Yunnan Lincang (E8), represented the target environments well. The overall ecological homogeneity was satisfactory and the test results were thus well targeted and of good representativeness. Two pilot sites, Guangxi Laibin (E4) and Yunnan Dehong (E7), represented the test environment relatively poorly.

### 3-D biplot analysis

**Figure 4:**
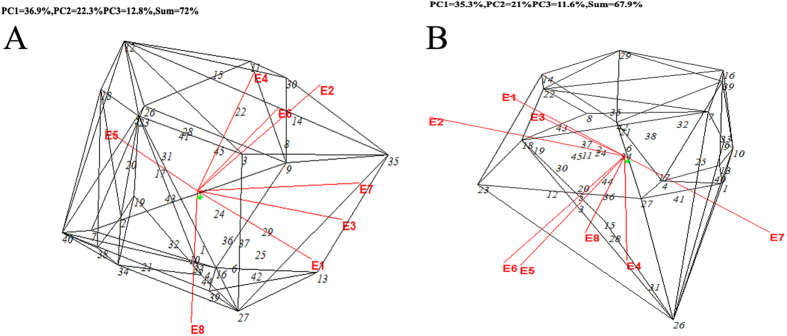
A 3D-GGE biplot analysis. Refer to Fig. 1 for abbreviations.

Due to the large number of cultivars tested, the information ratios (IR) of the third principle components for cane and sucrose yields were 1.024 and 0.928, respectively (Table 3). A 2-D biplot is not sufficient to show all the characteristics of the data. Nevertheless, the goodness of fit of the first three principle components in the 3-D biplot for cane and sucrose yields reached 72% and 67.9%, respectively, to allow a characterisation of the data (Fig. 4). Based on the 3-D biplot shown in Fig. 4A, the cane yields of 15 cultivars (YZ 06–407, FN 40, LC 03–1137, FN 38, YZ 04–241, FN 41, YT 96–86, DZ 03–83, MT 01–77, FN 39, YT 60, LC 05–136, YZ 05–51, GT 29 and FN 42) exceeded the cane yield of ROC22. Among these cultivars, FN 41, FN 38, FN 42, FN 39, YZ 06–407 and LC 05–136 also had a relatively high yield stability, whereas MT 01–77, LC 03–1137, GT 29, YT 96–86, DZ 03–83, FN 40, YZ 05–51, YT 60 and YZ 04–241 had a relatively low yielding stability. From the perspective of sucrose yields, 16 cultivars (LC 03–1137, FN 41, FN 38, LC 05–136, MT 01–77, YZ 05–51, DZ 03–83, YZ 06–407, FN 39, YG 40, FN 42, YZ 05–49, GN 02–70, YZ 06–80, YZ 06–189 and YZ 04–241) exceed ROC22 (Fig. 4B). Of these cultivars, FN 42, GN 02–70, YZ 04–241, DZ 03–83, YZ 06–80, YZ 05–49, YZ 06–407 and FN 39 also had a relatively high sucrose yielding stabilities, whereas FN 41, MT 01–77, YZ 05–51, FN 38, YG 40, LC 03–1137, LC 05–136 and YZ 06–189 had relatively low sucrose yielding stabilities.

## Discussion

Forty-five sugarcane cultivars were evaluated for yield potential and yield stability in this study. These included 12 YZ- and one DZ-series cultivars from Yunnan, four LC- and six GT-series cultivars from Guangxi, nine FN- and one MT-series cultivars from Fujian, ten YT-series cultivars from Guangdong, one GN-series cultivar from Jiangxi, and one check cultivar ROC 22. These cultivars were developed recently by several Chinese sugarcane breeding institutes and are highly representative products of the sugarcane breeding efforts in China. In addition, eight pilot test sites were chosen from the major sugarcane production areas for evaluation and demonstration purposes. Hence, the results of this study are of significance in guiding the selection and recommendation of superior sugarcane cultivars in China.

Nine cultivars, namely, YZ 06–407, LC 03–1137, FN 38, FN 41, DZ 03–83, MT 01–77, FN 39, LC 05–136 and YZ 05–51, produced both high cane and high sucrose yields as were predicted by the HA–GGE biplots. Five cultivars, namely, YG 40, YZ 06–189, YZ 05–49, YZ 06–80 and GN 02–70, produced relatively high sucrose yields, but lower cane yields compared to the check ROC 22. Cultivars GT 29, YT 60, FN 40 and YT 96–86 produced relatively high cane yields, but lower sucrose yields. These 18 cultivars had either cane or sucrose yield exceeding that of ROC22, and therefore were recommended for planting using a reasonable distribution of sugarcane cultivars based on local conditions.

Because of local conditions in China, an ideal sugarcane cultivar would have high and stable yield and would adapt to a wide range of environments^36^,^37^,^38^. In the present study, only four cultivars, namely, YZ 06–407, FN 39, YZ 05–51 and LC 05–136, fit that definition by producing higher and more stable cane and sucrose yields than the check cultivar ROC22. Two other cultivars, FN 38 and FN 41, produced higher cane and sucrose yields than ROC22 with a relatively high stability on cane yield but a relatively low stability on sucrose yield. Still, two other cultivars, LC 03-1137 and DZ 03–83, produced higher cane and sucrose yields than ROC22 with a relatively low stability on cane yield but a relatively high stability on sucrose yield. These result are consistent with a previous report by Luo *et al.*^31^, who also concluded that cultivar FN 39 was of high cane and sucrose yields as well as being generally stable. The fore-mentioned eight sugarcane cultivars are of high production value and are being recommended as widely adaptable cultivars for different trial stations.

Assessment of test environments in a target region is an important step during sugarcane cultivar trials^28^. HA-GGE biplot-based assessments can directly reveal the yield and stability properties of the cultivar as well as the discriminating power of test sites^24^,^26^,^27^,^28^,^29^,^30^,^31^,^34^. This helps sugarcane breeders identify test sites of good discriminating power and improve the accuracy of varietal trials^29^,^30^,^31^,^33^,^34^,^36^,^37^,^38^,^39^,^40^. Test sites that were selected based on a good discriminating power and a good representativeness of all test sites in the target region were proven to be ideal sites^39^. In the current study, the HA-GGE biplot was applied in preliminary assessments of eight pilot test sites. When taking both the cane and the sucrose yields into account, six pilot sites, namely, Guangdong Zhanjiang (E1), Guangxi Baise (E2), Guangxi Chongzuo (E3), Guangxi Liuzhou (E5), Hainan Lingao (E6) and Yunnan Lincang (E8) were shown to be highly representative of their target environments. The overall ecological homogeneity of these sites was satisfactory; hence, the test results were representative of these six pilot test sites. In contrast, two pilot test sites, Guangxi Laibin (E4) and Yunnan Dehong (E7), were not representative of their test environments.

How to assess both yield and yield stability of newly released sugarcane cultivars is a difficult research topic. Nevertheless, this information is very critical for guiding the rational distribution of sugarcane cultivars, the purpose of sugarcane breeding research. By using the HA-GGE biplot, ten newly released sugarcane cultivars, namely, DZ 03–83, FN 38, FN 39, FN 40, FN 41, LC 03–1137, LC 05–136, MT 01–77, YZ 05–51 and YZ 06–407, produced both higher cane and sucrose yields than the check cultivar ROC22. Eight cultivars are being recommended for rational distribution based on local conditions. Three cultivars were recommended for commercial production in the three dominant sugarcane production areas in China, namely, the Central and Southern Guangxi (LC 05–136), the Southwestern Yunnan (YZ 05–51), and the Western Guangdong and Northern Hainan (FN 39). Cultivars DZ 03–83 and YZ 06–407 were recommended for commercial production in all sugarcane plant areas except for Guangxi Chongzuo and Hainan Lingao. Cultivars MT 01–77, FN 38 and FN 41 were recommended for commercial production in Guangdong Zhanjiang, Guangxi Baise and Guangxi Chongzuo. LC 03–1137 was recommended for production in Guangxi Liuzhou, Hainan Lingao, and Yunnan Lincang. LC 03–182 was recommended for production in Guangxi Laibin and Yunnan Dehong. These results are of significance in guiding sugarcane breeding and rational regional distribution of sugarcane cultivars in China.

## Methods

### Ethics Statement

We confirm that no specific permits were required for the described locations/activities. We also confirm that the field studies reported did not involve endangered or protected species.

### Pilot sites

The field evaluation trials were conducted at the field pilot sites of eight sugarcane integrative experiment stations located in eight counties (or cities) of four provinces (synonymous with autonomous regions for this paper) in China, namely, Guangxi, Yunnan, Guangdong, and Hainan. These pilot sites were designated as GDZJ (E1) in Guangdong Zhanjiang, GXBS (E2) in Guangxi Baise, GXCZ (E3) in Guangxi Chongzuo, GXLB (E4) in Guangxi Laibin, GXLZ (E5) in Guangxi Liuzhou, HNLG (E6) in Hainan Lingao, YNDH (E7) in Yunnan Dehong, and YNLC (E8) in Yunnan Lincang. The elevation, latitude, longitude, precipitation, soil type, and other environmental parameters of these pilot sites^36^ are described in Table 4 .

### Sugarcane varieties

Forty-four newly released sugarcane cultivars from eight sugarcane research institutes (SRIs) were evaluated. These included 1 = YZ 99–91; 2 = YZ 06–80; 3 = YZ 06–407; 4 = YZ 05–51; 5 = YZ 05–49; 6 = YZ 04–241; 7 = YZ 03–422; 8 = YZ 03–258; 9 = YZ 03–194; 10 = YZ 03–103; 11 = YZ 01–1413; 12 = YZ 06–189; 13 = YT 96–86; 14 = YT 60; 15 = YT 55; 16 = YT 00–318; 17 = YT 00–236; 18 = YG 42; 19 = YG 40; 20 = YG35; 21 = YG 34; 22 = YG 24; 23 = MT 01–77; 24 = LC 05–136; 25 = LC 05–129; 26 = LC 03–182; 27 = LC 03–1137; 28 = GT 97–69; 29 = GT 30; 30 = GT 29; 31 = GT 02–901; 32 = GT 02–467; 33 = GT 02–351; 34 = GN 02–70; 35 = FN 40; 36 = FN 39; 37 = FN 38; 38 = FN 36; 39 = FN 30; 40 = FN 28; 41 = FN 15; 42 = FN 41; 43 = FN 42 and 44 = DZ 03–83. Another cultivar, ROC22( = 45), has been a dominant sugarcane cultivar in China and was served as the check variety in this study. The eight SRIs are located in five provinces (Guangxi, Yunnan, Guangdong, Fujian and Jiangxi), namely, the SRI of Yunnan Academy of Agricultural Sciences; Dehong SRI, Yunnan; Liucheng SRI, Guangxi; the SRI of Guangxi Academy of Agricultural Sciences, Guangxi; the SRI of Fujian Agriculture and Forestry University, Fujian; the Guangzhou Sugarcane Industry Research Institute, Guangdong; the Jiangxi SRI, Jiangxi; and the SRI of Fujian Academy of Agricultural Sciences, Fujian.

### Experimental methods

Test plot sites were selected that had flat terrain with uniform but representative local fertility levels. All had a convenient irrigation facility and grew the same crop prior to the test. All were at a considerable distance from tall buildings. A randomized block design was used with three replications. Each block had four 8–meter long rows with 1.1-meter spacing. Planting started from late February to early March at a rate of 105,000 cutting per hectare. Field management levels were the same as those for the local commercial fields. Cultivation, plowing, fertilization, irrigation and pest control were carried out on time. For field operations, the same technical measures were completed at the same pilot sites on the same day. Trials were conducted for two consecutive years to include data from two plant-cane and the first ratoon crops. The sugarcane crops were harvested in March of the following year. Before harvest, yield estimates were obtained as follows: the stalks in the two middle rows, the experimental rows, central row were manually harvested and mechanically weighed; the actual harvest area was measured, and the number of stalks harvested was counted. The cane yield per unit area was then calculated based on the area, number of stalks, and stalk weight. In the middle of each month from November to March of the following year, six millable stalks were collected and sucrose content was measured using the polarimetry method^37^. Sucrose yield was then calculated based on average sucrose content and cane yield using Equation (1): Sucrose yield (t ha^–1^) = Cane yield (t ha^–1^) × Sucrose content (%)

### Data analysis

The GGE-Biplot software was used for HA-GGE biplot analysis^26^,^40^. The cane yield data obtained from all pilot sites were summarized into a cultivar-plot site two-way table, and the average trait value of the corresponding cultivar at the corresponding trial site was taken as each value. The general model for GGE biplots is^26^,^40^:


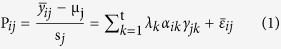


The response value (G) observed in target environment j’ due to indirect selection in test environment j is calculated^26^,^40^:


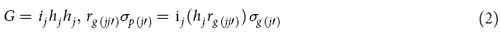


In the HA-GGE biplot model, the correlation between indices was evaluated by the cosine of the angle between adjacent indices. Starting from one index vector the cosine of the angle between the other index vector and the starting index vector is the correlation coefficient of two indices. The position of projection of the sugarcane cultivar or pilot site on the average-tester (AT) axis is used to assess the average performance of the cultivar or the representativeness of the site. The length of the projection of the sugarcane cultivar or plot site on the AT axis is used to determine the stability of the cultivar or the discriminating power of the plot site^30^. In the “appropriate combination of genotype and environment” in a functional diagram of the HA-GGE biplot, if the icons of the outmost cultivars are connected to form a polygon, the icons of all cultivars will be enclosed within the polygon. If a line is perpendicular to each side of the icons, all cultivars will be enclosed within the polygon. If a line perpendicular to each side of the polygon is drawn from the origin of the biplot, the polygon will be divided into different sectors, and all pilot sites within the same sector form a test site combination. Within each sector, the cultivar located at the polygon vertex is the cultivar with the best performance in all test sites of that sector, i.e., the best cultivar of that test site combination^30^.

To assess the test environment with a high accuracy, in the HA-GGE biplot the parameter used to assess the discriminating power of the test environment is the square root of the heritability, which approximately equals the length of the test environment vector. The parameter used to assess representativeness is the genetic correlation coefficient (*r*), which approximately equals the cosine of the angle between the test environment vector and the average environment axis. The ideal index of the test environment is the product of the square root of the heritability of the length of the vertical projection of test environment vector on the average environment axis and the genetic correlation coefficient *r*^26^,^27^,^28^,^29^,^30^.

Analysis of variance was conducted based on the general linear model for randomized complete blocks and the significance of various effects was based on F test against the experimental errors within environments^40^.

## Additional Information

**How to cite this article**: Luo, J. *et al.* Rational regional distribution of sugarcane cultivars in China. *Sci. Rep.* **5**, 15721; doi: 10.1038/srep15721 (2015).

## Figures and Tables

**Table 1:** Analysis of variance and basic statistics

Variation Source	Cane yield				Sucrose yield			
	DF	SS	F	%	DF	SS	F	%
Genotype (G)	44	109791.89	12	13.35	44	2757.66	12	13.80
Environment (E)	7	358436.58	247.2	43.58	7	8904.74	247.2	44.56
G × E	308	274885.05	4.3	33.42	308	6142.64	4.3	30.74
Block (Environment)	16	79374.60	24	9.65	16	2180.76	24	10.91
Error	1816	376105.18			1816	8490.71		
Grand mean (Mg.ha^-1^)		98.65				14.26		
SE		14.39				2.16		
CV%		14.59				15.16		
LSD (5%)		23.55				3.54		
G/GGE		0.29				0.31		

Note: DF, degrees of freedom; SS, Stdev square; F, f-statistic; SE, Standard error; CV%, Coefficient of variation; LSD, Least significant difference.

**Table 2:** Cane and sucrose yields at different pilot test sites (Mg·ha^–1^).

Environment (E)*	Cane yield					Sucrose yield				
	Average	MAX	SE**	SD**	CV%**	Average	MAX	SE	SD	CV%
E1	77.90	97.44	11.51	13.35	14.78	10.63	14.82	1.62	1.69	15.26
E2	111.63	147.49	14.46	16.49	12.95	16.15	21.86	2.22	2.60	13.74
E3	114.31	156.47	16.60	18.93	14.52	16.57	20.75	2.49	2.24	15.05
E4	85.85	107.97	14.09	16.07	16.41	12.65	17.57	2.09	1.66	16.48
E5	82.74	111.58	17.14	19.69	20.71	11.78	16.57	2.50	2.61	21.20
E6	100.47	119.49	10.25	11.71	10.20	13.50	16.96	1.44	1.38	10.65
E7	98.99	138.41	14.66	17.00	14.81	14.63	18.71	2.00	2.34	13.66
E8	96.95	137.30	15.06	17.46	15.53	14.98	22.43	2.64	3.14	17.65

^*^E1 = GDZJ, Guangdong Zhanjiang; E2 = GXBS, Guangxi Baise; E3 = GXCZ, Guangxi Chongzuo; E4 = GXLB, Guangxi Laibin; E5 = GXLZ, Guangxi Liuzhou; E6 = HNLG, Hainan Lingao; E7 = YNDH, Yunnan Dehong; E8 = YNLC, Yunnan Lincang

^**^SE, Standard error; SD, Standard deviation; CV%, Coefficient of variation.

**Table 3:** Eigenvalue, percentage of total variance explained, and information ratio (IR) of the first six principal components (PC).

PC	Cane yield			Sucrose yield		
	Eigenvalue	%SS	IR	Eigenvalue	%SS	IR
1	9.366	36.9	2.952	9.13	35.3	2.824
2	7.275	22.3	1.784	7.029	21	1.68
3	5.528	12.8	1.024	5.241	11.6	0.928
4	4.947	10.3	0.824	4.306	7.9	0.632
5	3.346	4.7	0.376	3.075	4	0.32
6	2.817	3.3	0.264	2.747	3.2	0.256

Note: SS, Stdev square.

**Table 4:** Environmental description of the eight pilot test sites.

Pilot site	Code	Longitude(E)	Latitude(N)	Altitude(m)	Soil type	Precipitation (mm)	Annual day length (h)	Mean annual temperature (^o^C)
GDZJ	E1	110.26	21.16	22	Red loam	1691	2106	23
GXBS	E2	106.98	23.68	82.5	Sandy soil	1100	1448	21
GXCZ	E3	108.55	22.94	78	Loam soil	1200	1600	20.8
GXLB	E4	109.08	23.76	95	Sandy soil	1400	1750	20.8
GXLZ	E5	109.36	24.47	99.1	Yellow soil	1700	1570	20
HNLG	E6	109.69	19.92	20	Red loam	1417	2349	24.5
YNDH	E7	97.85	24.01	780	Loam soil	1355	2330	21.2
YNLC	E8	99.95	24.15	1030	Red loam	1200	2257	19.6

Note: GDZJ, Guangdong Zhanjiang; GXBS, Guangxi Baise; GXCZ, Guangxi Chongzuo; GXLB, Guangxi Laibin; GXLZ, Guangxi Liuzhou; HNLG, Hainan Lingao; YNDH, Yunnan Dehong; YNLC, Yunnan Lincang.

## References

[b1] Liu X. X., Chen R. K.& Zheng C. F. Changes of Chinese sugar market under the background of changing for 63 years. Sugar Crops China 1, 68–70 (2013).

[b2] Chen R. K. *et al.* Modern sugarcane genetics and breeding. In: Chen R. K. editor. Beijing: China Agriculture Press. pp. 1–3 (2010).

[b3] Gauch H. G. & Zobel R. W. AMMI analysis of yield trails. In: Kang M. S. & Gauch G. H. ed. Genotype-by-Environment Interaction. Boca Raton Florida: CRC Press, pp 85–122 (1996).

[b4] Romagosa I. & Fox P. N. Genotype × environment interaction and adaptation. In: Hayward M. D., Bosemark N. O. & Romagosa I. ed. Plant Breeding: Principle and Prospects. London: Chapman and Hall, pp373–390 (1993).

[b5] Epinat-Le Signor C., Dousse S., Lorgeou J. & Denis J.-B. Interpretation of genotype × environment interactions for early maize hybrids over 12 Years. Crop Sci. 41, 663–669 (2001).

[b6] Rharrabti Y., Garcia del Mora l L.F., Villegas D. & Royo C. Durum wheat quality in Mediterranean environments III. Stability and comparative methods in analyzing G × E interaction. Field Crop Res. 80, 141–146 (2003).

[b7] Chang L.& Chai, S.X. Application of AMM I model in the stability analysis of spring wheat in rainfed area. Acta Ecol. Sin. 26, 3677–3684 (2006).

[b8] Giauffret C., Lothrop J., Dorvillez D., Gouesnard B.& Derieux M. Genotypexenvironment interactions in maize hybrids from temperate or highland tropical origin. Crop Sci. 40, 1004–1012 (2000).

[b9] Yan W. Biplot analysis of incomplete two-way tables. Crop Sci. 53, 48–57 (2013).

[b10] Gravois K. A. & Bernhardt J. L. Heritability and genotype× environment interactions for discolored rice kernels. Crop Sci. 40, 314–318 (2000).

[b11] Thomaszum F., Heiko C., Becker & Christian M. Genotype x environment interactions, heritability, and trait correlations of sinapate ester content in winter rapeseed (*Brassica napus* L.). Crop Sci. 46, 2195–2199 (2006).

[b12] CrossRef Gunasekera C. P., Martin L. D., Siddique K. H. M. &Walton G. H. Genotype by environment interactions of Indian mustard (*Brassica juncea* L.) and canola (*B. napus* L.) in Mediterranean-type environments 1. Crop growth and seed yield. *Europ*. J. Agron. 25, 1–12 (2006).

[b13] Luo J., Yuan Z. N., Zhang H., Chen Y. Q. & Chen R. K. Stability analysis on yield characters of sugarcane ratoon. Chin. J. Appl.& Environ. Biol. 15, 488–494 (2009).

[b14] Casanoves F., Baldessari J.& Balzarini M. Evaluation of multi-environment trials of peanut cultivars. Crop Sci. 45, 18–26 (2005).

[b15] Foucteau V., Daouk M. & El Bari C. Interpretation of genotype by environment interaction in two sunflower experimental networks. Theor. Appl. Genet. 102, 327–334 (2001).

[b16] Francis W., Wilson N. & Joseph O. Genotype × environment interactions for tea yields. Euphytica. 127, 289–296 (2002).

[b17] Walter R. F., Joseph A. H. & Susan L. J. Genotype and environment influence on protein components of soybean. Crop Sci. 43, 511–514 (2003).

[b18] Grüneberg W. J., Manrique K., Zhang D. P. & Hermann M. Genotype x environment interactions for a diverse set of sweet potato clones evaluated across varying ecogeographic conditions in peru. Crop Sci. 45, 2160- 2171 (2005).

[b19] Sabaghnia N., Oehghani H.& Sabaghpour S. H. Nonparametric methods for interpreting genotype × environment interaction ofLentil genotypes. Crop Sci. 46, 1100–1106 (2006).

[b20] Kang M. S. & Miller J. D. Genotype × environment interactions for cane and sugar yield and their implications in sugarcane breeding. Crop Sci. 24, 435–440 (1984).

[b21] Jackson P., McRae T. & Hogarth M. Selection of sugarcane families across variable environments II. Patterns of response and association with environmental factors. Field Crop Res. 43, 119–130 (1995).

[b22] Zhou M., Joshi S., Maritz T. & Koberstein H. Components of genotype by environment interaction among SASRI regional breeding and selection programmes and their implications. Proc. S African Sugar Technol. Assoc. 84, 363–374 (2011).

[b23] Hugh G. O. Statistical analysis ofyield trials by AMMI and GGE. Crop Sci. 46, 1488–1500 (2006).

[b24] Yan W., Kang M. S., Ma B. L., Woods S. & Cornelius P. L. GGE biplot vs. AMMI analysis of genotype-by-environment data. Crop Sci. 47, 643−655 (2007).

[b25] Bissessur D., Chong L. C., Ramnawaz C. & Ramdoyal K. Analysing G × E interaction in sugar cane using the Additive Main Effects and Multiplicative Interaction (AMMI) Model. Proc. Int. Soc. Sugar Cane Technol. 24, 506–511 (2001).

[b26] Yan W. K. & Hant L. A. Biplot analysis of diallel data. Crop Sci. 42, 21–30 (2002). 11756249 10.2135/cropsci2002.0021

[b27] Yan W. K. Methodology of cultivar evaluation based on yield trial data with special reference to winter wheat in Ontario. PhD Thesis, University of Guelph, Guelph, Ontario, Canada (1999).

[b28] Yan W. K., Hunt L. A., Sheng Q. & Szlavnics Z. Cultivar evaluation and mega-environment investigation based on the GCE biplot. Crop Sci. 40, 597–605 (2000).

[b29] Yan W. K. GGE biplot-a Windows application for graphical analysis of multi-environment trial data and other types of two way data. Agron. J. 93, 1111–1118 (2001).

[b30] Yan W. K. Optimum use of biplots in the analysis of multi−environment variety trial data. Acta Agron. Sin. 36, 1−16 (2010).

[b31] Luo J. *et al.* Analysis of yield and quality traits in sugarcane cultivars (lines) with GGE-Biplot. Acta Agron. Sin. 39, 142–152 (2013).

[b32] Zhou C. J., Tian Z. Y. & Li J. Y. GGE-Biplot analysis on yield stability and testing-site representativeness of soybean lines in multi-environment trials. Soybean Sci. 30, 318−322 (2011).

[b33] Zhang Z. F., Fu X. F., Liu J. Q. & Yang H. S. Yield stability and testing-site representativeness in national regional trials for oat variety based on GGE-Biplot analysis. Acta Agron. Sin. 36, 1377–1385 (2010).

[b34] Xu N. Y., Zhang G. W., LI J. & Zhou Z. G. Evaluation of cotton regional trial environments based on HA-GGE biplot in the Yangtze river valley. Acta Agron. Sin. 38, 2229–2236 (2012).

[b35] Xu N. Y., Zhang G. W., LI J. & Zhou Z. G. Ecological regionalization of cotton varieties based on GGE biplot. Chin. J. Appl. Ecol. 24, 771–776 (2013). 23755494

[b36] Luo J. *et al.* Evaluation of sugarcane test environments and ecological zone division in China based on HA-GGE biplot. Acta Agronomica Sinica 41, 214–227 (2015).

[b37] Luo J., Zhang H., Deng Z. H. & Que Y. X. Trait stability and test site representativeness of sugarcane cultivars based on GGE-biplot analysis. Chin. J. Appl. Ecol. 23, 1319–1325 (2012). 22919843

[b38] Luo J., Pan Y.-B., Xu L. P., Zhang H. & Yuan Z. N. Cultivar evaluation and essential test locations identification for sugarcane breeding in China. Sci. World J. 2014, Article 302753 (2014). 10.1155/2014/302753 PMC4055468 24982939

[b39] Luo J., Que Y. X., Zhang H. & Xu L. P. Seasonal variation of the canopy structure parameters and its correlation with yield-related traits in sugarcane. Sci. World J. 801486 (2013). 10.1155/2013/801486 PMC3888768 24453909

[b40] Yan W. & Holland J. B. A heritability-adjusted GGE Biplot for test environment evaluation. Euphytica 171, 355–369 (2010).

